# Fall Tailoring Interventions for Patient Safety Brazil Program: an evaluability study in a teaching hospital

**DOI:** 10.1590/0034-7167-2023-0348

**Published:** 2024-05-27

**Authors:** Adeli Regina Prizybicien de Medeiros, Luciana Schleder Gonçalves

**Affiliations:** I Universidade Federal do Paraná. Curitiba, Paraná, Brazil

**Keywords:** Program Evaluation, Patient Safety, Accidental Falls, Hospitals Teaching, Implementation Science, Evaluación de Programas y Proyectos de Salud, Seguridad del Paciente, Accidentes por Caídas, Hospitales de Enseñanza, Ciencia de la Implementación, Avaliação de Programas e Projetos de Saúde, Segurança do Paciente, Acidentes por Quedas, Hospital de Ensino, Ciência da Implementação

## Abstract

**Objectives:**

to present the theoretical model, logic model, and the analysis and judgment matrix of the Fall TIPS Brazil Program.

**Methods:**

a qualitative, participatory research approach, in the form of an evaluability study, encompassing the phases (1) problem analysis; (2) program design, development, and adaptation to the Brazilian context; (3) program dissemination. Data were collected through document analysis and workshops.

**Results:**

through document analysis, workshops with stakeholders from the participating institution, and validation with key informants, it was possible to identify the program’s objectives, expected outcomes, and the target audience. This allowed the construction of theoretical and logic models and, through evaluative questions, the identification of indicators for the evaluation of the Fall TIPS Brazil Program.

**Final Considerations:**

this study has provided insights into the Fall TIPS program, the topic of hospital fall prevention, and the proposed models and indicators can be employed in the implementation and future evaluative processes of the program.

## RESUMO

**Objetivos:**

apresentar o modelo teórico, modelo lógico e matriz de análise e julgamento do programa *Fall* TIPS Brasil.

**Métodos:**

pesquisa qualitativa, participativa do tipo estudo de avaliabilidade, contempla as fases (1) análise do problema; (2) design, desenvolvimento e adaptação do programa à realidade brasileira; (3) disseminação do programa. Os dados foram coletados por análise documental e oficinas.

**Resultados:**

a partir da análise documental, oficinas com *stakeholders* da instituição participante e validação junto a informantes-chave, foi possível identificar os objetivos do programa, os resultados esperados e o público beneficiado, construir os modelos teórico e lógico e, mediante perguntas avaliativas, apontar indicadores para a avaliação do programa *Fall* TIPS Brasil.

**Considerações Finais:**

o estudo trouxe conhecimentos sobre o programa *Fall* TIPS, sobre a temática de prevenção de quedas hospitalares, e os modelos e indicadores propostos podem ser empregados na implantação e em processos avaliativos futuros do programa.

## RESUMEN

**Objetivos:**

presentar el modelo teórico, modelo lógico y matriz de análisis y juicio del programa *Fall* TIPS Brasil.

**Métodos:**

investigación cualitativa, participativa tipo estudio de evaluabilidad, que abarca las fases (1) análisis del problema; (2) diseño, desarrollo y adaptación del programa a la realidad brasileña; (3) difusión del programa. Los datos se recopilaron mediante análisis documental y talleres.

**Resultados:**

a través del análisis documental, talleres con partes interesadas de la institución participante y validación con informantes clave, se pudieron identificar los objetivos del programa, los resultados esperados y el público beneficiario, construir los modelos teórico y lógico y, a través de preguntas evaluativas, señalar indicadores para la evaluación del programa *Fall* TIPS Brasil.

**Consideraciones Finales:**

el estudio aportó conocimientos sobre el programa *Fall* TIPS, sobre la temática de prevención de caídas en hospitales, y los modelos e indicadores propuestos pueden ser utilizados en la implementación y en futuros procesos de evaluación del programa.

## INTRODUCTION

Falls among hospitalized patients are frequent and complex patient safety incidents, with an increased concern due to their potentially preventable nature^(1)^ through the implementation of multimodal strategies involving patients, caregivers, and the multidisciplinary team^(1, 2)^. Additionally, they are often associated with increased healthcare costs^(3, 4)^. In Brazil, they rank among the most frequently reported incidents, with historical data from 2014 to 2022 demonstrating their prevalence^(5)^.

In response to the need to address this situation, the Fall Tailoring Interventions for Patient Safety (Fall TIPS) is a fall prevention program conceived in the United States, resulting from over a decade of research and adopted in more than 200 hospitals worldwide. It consists of a portfolio of scientifically anchored tools for the consistent assessment of fall risks, shared proposition, and implementation of a personalized plan for fall prevention. It emphasizes the engagement of managers, leaders, champions, regarded as multipliers, as well as healthcare professionals, patients, and caregivers^(6)^. Although these individuals are originally referred to as stakeholders in the original program, in this study, they are referred to as “agents” due to the use of the term “stakeholders” in the adopted methodology.

The tools of the Fall TIPS program include a laminated colored poster for bedside placement or attachment to an electronic monitor; computerized modules to be incorporated into the electronic medical record, as well as instruments and educational materials that guide its implementation and monitoring^(1)^. In Brazil, the program has been in the process of implementation since 2019, during which cross-cultural adaptation, educational material production, and the development of theoretical and logical models have taken place. Additionally, a website has been created for the allocation and dissemination of these resources^(7)^.

Most programs do not have theoretical and logical models representing them before their implementation^(8)^. To address this gap, the interest of academia and healthcare professional practice settings in Brazil in adapting Fall TIPS to the Brazilian context, as is already happening in other countries worldwide, paved the way for the conduct of an Evaluability Study (ES), also known as a situation analysis, strategic analysis, and logical analysis^(8)^. ES, consisting of a set of preparatory procedures for evaluation, is relevant at any stage of its development, especially with the participation^(9)^ of stakeholders, understood as supporters and interested parties whose decisions can affect the program’s future and add knowledge that can maximize the chances of using the results of a future evaluation^(9, 10, 11)^. The theoretical and logical models^(10, 12)^ are essential communication tools for understanding the components, resources, activities, and results attributable to a program^(13, 14)^. They also facilitate the construction of indicators useful for conducting future evaluation processes.

## OBJECTIVES

To present the theoretical model, logical model, and the analysis and judgment matrix of the Fall TIPS Brazil program.

## METHODS

### Ethical considerations

The study was conducted in accordance with national research ethics guidelines and received approval from the Research Ethics Committee with Human Subjects at the Hospital de Clínicas Complex of the Federal University of Paraná.

### Study design

This research is a qualitative, participatory study^(15)^ characterized as an evaluability study^(11, 16)^. It is part of the second phase of the project titled “Dissemination and Adoption of Fall TIPS Brazil: Engaging Patients, Professionals, and Clinical Leadership for Fall Prevention in the Hospital Setting.” This project spans four phases from February 2020 to July 2023: (1) problem analysis; (2) design, development, and adaptation of the program to the Brazilian context; (3) program dissemination. Phase 4, which involves implementation and subsequent evaluation, is scheduled for the period from 2024 to 2026.

### Methodological procedures

Chart 1 presents the components of the Evaluability Study (ES) along with their respective objectives and strategies

**Chart 1:** Components, Objectives, and Strategies of the Evaluability Study

Components of the Evaluability Study^(11, 16)^	Objectives	Strategies
1. Definition of the ES’s focus.	Define the focus of the ES.	Analysis of documents, reports, and scientific publications on patient safety, fall prevention, and the Fall TIPS program, conducted by the researcher, from August 2021 to July 2023. Conducting six meetings with institution managers, preceding the request for the identification of stakeholders from the nursing managers and the Quality and Patient Safety Management Department.
2. Development of an initial program theory.	Identify the stakeholders of the program in the researched healthcare institution.	Conducting six workshops^(17)^ by the researcher with the participation of stakeholders: Each lasting 60 minutes, held from September to November 2022. Audio recording with prior authorization. Utilization of a planner. Included the presence of observers who are part of the Fall TIPS Brazil project.
3. Gathering feedback on the program theory.	Construct the theoretical and logical models and propose indicators for the evaluation of the Fall TIPS Brazil program.	Conducting three validation workshops^(17)^ by the researcher, two online and one in-person: Each lasting 90 minutes, held from June to July 2023, under the researcher’s guidance. Audio recording with prior authorization.
4. Contributions to the uses of the ES.	4Validate the theoretical model, logical model, and indicators for the Fall TIPS Brazil program.	Preparation of a synthesis on the uses of the ES by the researcher in July 2023.

*ES – Evaluability study; Fall TIPS – Fall Tailoring Interventions for Patient Safety.*

### Study Setting

A public teaching hospital in Southern Brazil has been actively involved in patient safety and quality management activities for over a decade through its Quality and Patient Safety Management Department and collaborations with academia.

### Data Source

The stakeholders who took part in components one and two included 15 healthcare professionals selected through intentional non-probabilistic sampling, based on recommendations from hospital management. Their contributions contributed to the development of an initial version of the models, which was deemed suitable for the subsequent component, during which key informants further elaborated and refined the materials.

Inclusion criteria encompassed professionals working in healthcare, management, and/or clinical risk management fields, as well as those with technical and leadership responsibilities, irrespective of their professional category. Recruitment occurred through invitations extended by nursing managers and individuals responsible for quality and patient safety management at the participating institution, following a pre-established schedule for workshop implementation.

Conversely, four key informants who partook in component three were chosen based on their roles in teaching and/or management within the patient safety field or their experience in evaluating healthcare services and their prior knowledge of the Fall TIPS program. They were recruited via email invitations. For all participants, the exclusion criterion was their absence from academic, teaching, managerial, or clinical activities during the data collection period, due to various constraints.

### Data Collection and Organization

In component one, which focused on defining the scope of the ES, data collected from documents and publications were organized to address key points necessary for the development of the theoretical and logical models^(18)^. These data were then recorded in electronic spreadsheets, which were used to create flowcharts containing the information required for constructing the theoretical and logical models of the Fall TIPS Brazil program. This material was discussed among the research team members with the aim of generating ideas and improving the diagrams of the theoretical and logical models.

In components two and three, workshops were conducted based on the 4 Rs of Convergent Care Research^(17)^, namely: “Rethink”, “Recognize”, “Reveal”, and “Share”. With the exception of the first workshop, which started with “Recognize/Reveal”, each workshop commenced with “Rethink”. The previous workshop’s material and suggestions for changes resulting from previous discussions were projected for discussion. This was followed by a new discussion of the theme until consensus was reached. Subsequently, the “Recognize/Reveal” stage involved a brief presentation of the day’s topic, followed by the “Share” stage, aimed at facilitating discussion, providing a brief summary, communication, and expressions of gratitude.

In component two, which focused on developing an initial theory of the program, data were collected through the use of an individualized tool called a “planner”. This “planner” contained a QR code for electronic access to materials on the Brazilian program website^(7)^. Additionally, the “planner” included a preliminary version of the theoretical and logical models, incorporating open-ended questions and 26 evaluative questions suggested by the literature^(16)^. These questions were presented on a 10-point Likert Scale, where one signified the highest importance and ten represented the lowest. The “planner” also provided space for the inclusion of new questions. During two workshops, two rounds were conducted to prioritize the importance of the evaluative questions. After each round, responses were transcribed, and data were organized in electronic spreadsheets to calculate relative frequencies. A minimum consensus of 20% was targeted to identify priority questions. Participant responses in the “planners” were labeled with alphanumeric coding from Participant (P) one through 15, and these responses were organized in electronic spreadsheets. Discussions were recorded using audiovisual resources, transcribed using text editors, and assigned the same coding as the “planners”. The outcome yielded an initial version of the models and two evaluative questions that reached consensus.

In component three, which involved the collection of feedback on the program theory, at the outset of the first workshop with key informants, printed versions of the products from component two were distributed. These materials were supplemented with a proposal for indicators to evaluate the Fall TIPS Brazil program. Discussions were recorded using audiovisual equipment, coded (Informant(I) to I3), and transcribed using text editors. Component four, which included contributions to the uses of the ES, entailed synthesizing the findings from the previous components. All components were conducted by the researcher, a nurse, a patient safety specialist, and a nursing doctoral student who has been professionally engaged at the participating institution since 2004.

### Data Analysis

For the data from component one of the ES, the key points used as a guide for developing the models included the following:

1. The problem situation that the program aims to address.
2. The healthcare program created to address this situation.
3. The overall goal of the program.
4. The specific objectives of the program.
5. The targets the program intends to achieve.
6. The target population of the program.
7. The dimensions of the program.
8. The activities conducted within the program.
9. The structures required for the program to fulfill its function.
10. The products expected to be obtained through the program’s implementation.
11. The results the program aims to achieve.
12. External factors that may influence the achievement of results, beyond those directly related to the program^(18)^.

After obtaining the textual corpus, which resulted from transcribing the “planners” and recording workshops with stakeholders and key informants (components two and three), a content analysis was performed^(19)^. The choice of this technique is justified by its qualitative nature, allowing for an in-depth analysis of subjective aspects, while recognizing the non-neutrality among the researcher, the research subject, and the context. The data were correlated and integrated into the theoretical and logical models, serving as the basis for proposing evaluation indicators, which were considered thematic categories.

## RESULTS

### Component one: defining the focus of the Evaluability Study

Document review provided answers to key questions^(18)^ regarding the theme of hospital falls and the Fall TIPS program, and the synthesis of the findings is presented in Chart 2.

**Chart 2:** Sources of information for the development of the theoretical and logical models of the Fall Tailoring Interventions for Patient Safety Brazil program and their correlation with key questions answered

Key Questions	Responses to Key Questions
1. Problem situation to be addressed by the program	Hospital falls, often caused by multiple factors, are preventable in over 90% of cases. Most of the time, they occur in areas adjacent to the patient’s bed and room, despite fall risk assessment and recommendations for universal prevention care^(20, 21, 22, 23, 24, 25, 26, 27, 28, 29)^.
2. Healthcare program created to solve the problem	Fall TIPS adapted to the Brazilian context^(28, 29, 30, 31, 32, 33, 34)^.
3. Overall goal of the program	To prevent falls and their associated harm in healthcare institutions through a laminated poster for bedside placement, available to adult patients, caregivers, and healthcare teams. This poster allows for the collaborative identification of individual risk factors and their correlation with fall prevention strategies^(30, 31, 32, 33, 34)^.
4. Specific objectives of the program	4a. To provide patient-centered care based on experiences related to fall prevention from the program^(30, 31, 32, 33, 34)^. 4b. To contribute to the dissemination of a safety culture among professionals, patients, and caregivers^(30, 31, 32, 33, 34)^. 4c. To facilitate effective communication among professionals, patients, and caregivers^(30, 31, 32, 33, 34)^. 4d. To develop skills and competencies in the healthcare team necessary for providing patient-centered care^(30, 31, 32, 33, 34)^.
5. Goals to be achieved by the program.	(5a) Establish norms, standards, and routines focused on fall prevention among hospitalized patients in healthcare institutions, from the planning, execution, evaluation, and monitoring of personalized preventive interventions for each patient^(28, 29, 30, 31, 32, 33, 34)^. (5b) Develop the work process of healthcare teams with a focus on patient-centered care^(28, 29, 30, 31, 32, 33, 34)^. (5c) Involve the patient/caregiver in fall prevention, from admission to discharge^(28, 29, 30, 31, 32, 33, 34)^.
6. The target population of the program.	The stakeholders include healthcare professionals, hospitalized patients, and caregivers^(20, 21, 22, 23, 24, 25, 26, 27, 28, 29, 30, 31, 32, 33, 34)^.
7. The dimensions of the program.	Resources are subdivided into financial, physical, material, and human resources; the care dimension encompasses champions, healthcare professionals, and patients/caregivers; the patient and caregiver engagement dimension; continuous education; and the management dimension^(30, 31, 32, 33, 34)^.
8. The activities carried out within the program.	The program involves fall risk assessment, the proposal and implementation of a care plan with the engagement of patients, caregivers, and healthcare professionals at all stages. It also includes the management of the change in the care process and the recruitment and training of healthcare professionals to participate in the program. Patient and caregiver engagement is essential^(27, 30, 31, 32, 33, 34)^.
9. Structures required by the program to fulfill its function.	The resources required for the program include a physical environment for the continuous education of healthcare professionals, financial resources, human resources, materials, and supplies needed for its implementation and sustainability, including the laminated poster for bedside placement^(30, 31, 32, 33, 34)^.
10. The expected products to be obtained through the program’s implementation.	Reduction in falls and their associated harm in hospitalized patients through person-centered care^(27, 30, 31, 32, 33, 34)^.
11. The results the program aims to achieve.	Positive evolution of the safety culture regarding co-production of care with patients and caregivers for a hospitalization free of falls^(27, 30, 31, 32, 33, 34)^.
12. Factors that may influence the achievement of results, beyond those directly related to the program.	a) Factors of internal/political/institutional context refer to factors related to management support in implementing the program, including the allocation of human, financial, physical, and material resources, as well as institutional culture^(27, 30, 31, 32, 33, 34)^. b) Factors of external context are related to the organization of healthcare systems, whether public or private, and are linked to the political, economic, and healthcare programs’ landscape of the country^(27, 30, 31, 32, 33, 34)^.

### Component Two: Development of an Initial Program Theory

In Component 2, all 15 nominated stakeholders participated in the study: 14 nurses, with 13 (92.5%) females and 1 (7.14%) male. One female professional, with a medical degree, was nominated due to her previous participation in continuous improvement cycles within the institution. Among the 15 stakeholders, the average age was 41.46 years, with six (40%) being under 40 years old. Their years of experience ranged from two to 27 years, with an average of 16.3 years. Regarding their highest degree, two had undergraduate degrees (13%), three had postgraduate specializations (20%), six had master’s degrees (40%), and four had doctoral degrees (27%). The length of time working in their current area ranged from less than one year to 15 years, with an average of six years. In terms of their area of expertise, two worked in patient safety, five were in nursing management and permanent education committees, and eight worked in units related to bone marrow transplants, infectology, surgical clinics, and maternal and child care.

We began with an initial proposal for a theoretical model of the program, which included a simple block diagram covering external and internal contexts, program implementation, and program effects. Regarding the diagram, stakeholders had differing opinions, with some expressing surprise and varying degrees of importance regarding the prevention of falls in hospitalized patients, as shown in the excerpts:

*Healthcare professionals need to understand or be aware of how the program’s implementation will contribute to reducing falls.* (P9)

[We need to] *strengthen the prevention of falls as one of the institutional goals* [...] *and encourage fall reporting* [...]. (P4)

In this context, participants offered suggestions about what would be necessary internally for the implementation of the Fall TIPS program, as shown in the excerpts:

*Change in institutional culture* [...]. (P1)

*Training and educating professionals* [...]. (P1, P2)

[...] *professionals “need” to understand or “make it clearer” the importance of patient and caregiver engagement.* (P13)

Regarding the logical model, the initial proposal had been organized based on the activities performed by each agent within the program. However, in the last workshop with stakeholders, a version was presented that consolidated all agents, but this version was considered unclear and lacking in information, as pointed out in the excerpt:

*The diagram* [of the logical model] *does not reflect the planning and implementation of the strategy* [Fall TIPS program], *and I couldn’t visualize its purpose* [...] *as it does not achieve its goal* [...]. (P6, P7, P10)

Regarding the evaluative questions contained in the planner, no additions were suggested by the stakeholders. The two questions that reached consensus and were given the highest priority were: 1) What needs does the program address and what needs should be addressed, with 44% consensus; 2) What are the perceived objectives of the program and what should be the objectives, with 33% consensus. However, it is important to note that two questions related to human resources and the availability of documents/data for program evaluation after its implementation reached lower priority, ranking 6th with 44% consensus and 10th with 33% consensus, respectively. It’s worth highlighting that these questions are more related to the need for gaining a deeper understanding of the program and the problem it aims to solve, which is falls in hospitals.

### Component Three: Gathering Feedback on the Program Theory

Out of the four key informants who were invited, three actively participated, all of them female, with an average age of 53.6 years and education ranging from 15 to 43 years, averaging 31.6 years. Two held doctoral degrees, and one had a master’s degree in nursing. In this phase, after crafting an initial program theory and incorporating changes proposed by the stakeholders, we obtained the theoretical model, the logical model, and a proposal for evaluation indicators, all of which were validated by the key informants.

The key informants concurred with the stakeholders regarding the lack of clarity and provided suggestions that led to the inclusion of a central figure in the theoretical model, thereby making the roles of different stakeholders in the Fall TIPS Brazil program more evident. Consequently, the term “Network Organization” was proposed to be added to the external context, offering a systemic perspective encompassing the internal environment of the healthcare institution. Furthermore, the external context encompassed both public and private healthcare service providers, both subject to Brazilian guidelines and legislation, as well as the prerogatives of the National Patient Safety Program (PNSP). In this context, it was acknowledged that organizing public and private services into networks is a reality that can promote safer healthcare practices.

Concerning the logical model, the key informants also recommended the inclusion of a glossary of terms and archetypes featuring animals from the Brazilian fauna to represent each agent in the program (developed in another stage of the larger project). Another suggestion involved incorporating the names of the tools and instruments that underwent translation and cross-cultural adaptation, covering the dimensions and activities of the program. Therefore, we decided to include the information “utilization of Fall TIPS tools and instruments” in the sub-dimensions.

Furthermore, the key informants proposed restructuring the model into two primary dimensions: the managerial process and the care process. The managerial process dimension encompassed sub-dimensions such as pre-implementation, implementation, and execution, including resources; actions for continuous education; planning and implementation activities; as well as those related to monitoring adherence and program outcomes. Conversely, the care process dimension, with a sub-dimension titled execution, incorporated elements like actions for recruiting/coopting program-supporting professionals; actions for engaging patients and caregivers, linked to risk assessment; development; and the execution of personalized fall prevention plans.

The key informants emphasized the role of safety culture as an outcome of the activities outlined in the logical model of the Fall TIPS program. Additionally, they raised two other themes: a1 the need to involve students in academic activities within the hospital as participants in the Fall TIPS program, particularly in teaching hospitals; and b1 the role of professionals involved in hospital discharges of patients at risk of falls. Finally, the anticipated impacts of the Fall TIPS Brazil program extended beyond the hospital setting and fall prevention, encompassing aspects related to society’s health, economy, and the healthcare system.

**Figure 1:**
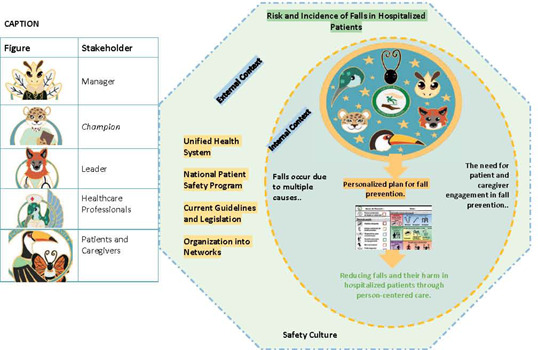
Theoretical Model of the Fall Tailoring Interventions for Patient Safety Program for Brazilian Hospitals

**Figure 2:**
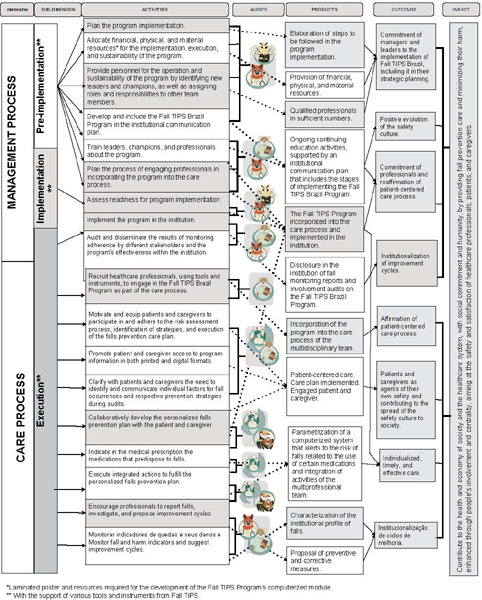
Logical Model of the Fall Tailoring Interventions for Patient Safety Program for Brazilian Hospitals

Consequently, these discussions led to the development of the theoretical model (Figure 1) and the logical model (Figure 2) of the Fall TIPS program for Brazilian hospitals.

The participants went beyond the previously selected evaluative questions in component two and suggested indicators related to the logical model based on their experiences with program implementation in other settings. These indicators were divided into two categories:

Managerial Process:

1. Percentage of compliance with the use of the laminated poster.
2. Percentage of healthcare professionals and leaders/champions who completed training on the Fall TIPS program.
3. Rate of falls per 1000 patients/day.
4. Rate of falls with harm per 1000 patients/day.
5. Percentage of healthcare team satisfaction with the Fall TIPS program.

Care Process:

1. Percentage of patients for whom the risk assessment was completed.
2. Percentage of prescription and implementation of the personalized fall prevention plan.
3. Percentage of patient and caregiver engagement in the Fall TIPS program.

These indicators encompass both the program’s effectiveness in preventing falls and the adherence and engagement of healthcare professionals, patients, and caregivers in program activities. They provide a comprehensive view of the performance and outcomes of the Fall TIPS Brazil program in Brazilian healthcare institutions.

### Component Four: Contributions to the Uses of the Evaluability Study

In summary, the study results indicate that the Fall TIPS Brazil program is amenable to evaluation, as theoretical and logical models have been developed and validated, along with the proposal of indicators for its assessment and monitoring. Given the innovative nature of the program in the country, this information can be valuable in planning evaluation approaches to address the needs that may arise during its implementation in different contexts.

## DISCUSSION

Developed in the 1980s and bolstered by criticisms of positivist social science, participatory approaches in the evaluation and pre-evaluation processes, such as EAs, are characterized by the inclusion of interest groups affected by a given intervention^(35, 36)^. Consequently, they underscore the importance of engaging stakeholders in defining the indicators to be assessed, ensuring that the evaluated results, which determine programmatic success, are relevant and meaningful within a specific context^(37)^.

In this manner, the definition of the study’s focus converged towards highlighting pathways for a better comprehension of the Fall TIPS program, its objectives^(38)^, and outcomes. These outcomes encompass a reduction of 15 to 25% in the number of falls among young patients^(1, 32)^, as well as a 34% decrease in falls resulting in harm among older patients^(1)^. Furthermore, the study’s focus addressed pertinent demands related to the specific implementation of the program in the Brazilian healthcare landscape.

Regarding the theoretical model, the elements identified in both internal and external contexts aligned with existing literature, particularly concerning the influence and reciprocity between them, and their relationships with the components, objectives, resources, activities, and effects of a program^(39)^. Similarly, the notation of a safety culture permeating both contexts^(40)^ was emphasized, given its intrinsic and cross-cutting nature. The external context encompasses social, political, and economic aspects external to the institution^(41)^, where the organization of public and private services into networks is a reality, and a factor capable of promoting safer healthcare practices. Likewise, in the Brazilian context, both spheres of service delivery are influenced by the National Patient Safety Program (PNSP), its regulations, and the Unified Health System (SUS), which is universal, public, decentralized in terms of responsibilities and resources, and inspired by values such as equality, democracy, and empowerment^(42)^.

From its conception to implementation in different realities, the creators of the Fall TIPS program refined processes to facilitate its adoption in different contexts^(34)^, based on the science of implementation. Beyond implementation, evaluating results, and defining program impacts, one of the challenges is to convert errors and successes from the change process into continuous learning within healthcare systems^(43)^. Thus, the availability of tools and instruments on the Fall TIPS website aligns with a critical characteristic during the implementation of evidence-based practices, namely, the potential for adaptations. To do so, these adaptations must be made systematically and faithfully adhering to the program’s principles^(44)^, with the awareness and collaboration of its creators^(34)^.

In EAs, the definition of evaluative questions follows the definition of what the program is, its objectives, goals, and how its components are articulated to achieve these objectives^(16)^. Therefore, it constitutes a crucial step in reflecting on the program^(45)^. In this sense, the discovery of differing understandings regarding the magnitude of falls in the studied environment suggests that attention should be given to the problem and the program with stakeholders throughout the implementation process. This measure can serve as a facilitating condition as it points to useful conceptual aspects^(46)^.

The implementation of evidence-based programs in different contexts is challenging, beginning with the time required to transform patient-reported outcomes and preferences into sustainable solutions^(47)^. Beyond reducing falls and their associated harm, it is understood that adopting the Fall TIPS program’s toolkit and documents will equip Brazilian professionals and healthcare services with technical and interpersonal skills beneficial for managing other patient safety incidents. This will be accomplished through the use of strategies to approach, encourage, and engage patients and caregivers, considered essential for promoting a culture of safe healthcare delivery^(48)^.

To establish roles throughout the implementation process, mapping stakeholders at all levels and sectors precedes the development of strategies and the estimation of the time dedicated to their involvement^(37)^. It is essential to note that executing complex projects is not devoid of surprises related to unforeseen issues that may introduce tensions and challenges during implementation^(49)^. In the interim, the importance of information gathered during the planning, implementation, and conclusion phases of projects or interventions is recognized as tools for program implementation^(49)^ and as frameworks for the continuation of evaluative research^(10)^. To enhance internal validity and study reliability, available scientific evidence was leveraged, including document analysis, workshops with stakeholders and informants, and a literature review primarily from the last 5 years.

### Study limitations

This research exhibits some limitations. The delay in the Fall TIPS Brazil project phases was attributed to the Covid-19 pandemic. The predominance of nursing-related stakeholders may suggest selection bias. Additionally, data collection during a pandemic period could have influenced the results. Another limitation is that in this study, the ES was utilized as a preparatory stage without the practical application of its products, which could be considered a limitation.

### Contributions to the Field

The primary contributions of this study to the nursing and patient safety field include the provision of theoretical and logical models for the Fall TIPS Brazil program, along with a proposal for indicators that extend beyond positivist evaluation perspectives. The implementation of the method, aligned with implementation science and including the dissemination of related products, facilitates a reduction in the time between the publication of new evidence and its implementation in various healthcare settings. The ES’s products will be accessible on the Fall TIPS Brazil program’s website and can be utilized by Brazilian healthcare organizations interested in implementing the program and conducting future evaluation processes within Brazilian healthcare institutions.

## CONCLUSIONS

In this ES, the program theory was developed, establishing the context and its logical framework, encompassing objectives, goals, activities, products, outcomes, and impact, elements that had not been systematically outlined in the literature and documents related to the Fall TIPS program. Simultaneously, it anticipated the challenges presented by the execution of complex projects based on participatory approaches, which do not always follow predetermined paths and necessitate flexibility, creativity, and receptiveness to adaptations by those involved.
